# Nicorandil Ameliorates Depression‐Like Behaviors After Traumatic Brain Injury by Suppressing Ferroptosis Through the SLC7A11/GPX4 Axis in the Hippocampus

**DOI:** 10.1002/brb3.70199

**Published:** 2024-12-31

**Authors:** Yao‐Ran Tu, Ming Tan, Yao Li, De‐Quan Hong, Fan Niu

**Affiliations:** ^1^ Department of Emergency and Trauma Center Nanchang First Hospital Nanchang Jiangxi China

**Keywords:** depression, ferroptosis, nicorandil, the SLC7A11/GPX4 axis, traumatic brain injury

## Abstract

**Introduction:**

Depression is a prevalent and significant psychological consequence of traumatic brain injury (TBI). Ferroptosis, an iron‐dependent form of regulated cell death, exacerbates the neurological damage associated with TBI. This study investigates whether nicorandil, a potassium channel opener with nitrate‐like properties known for its antioxidative and neuroprotective effects, can mitigate depression‐like behaviors following TBI by modulating ferroptosis.

**Methods:**

A controlled cortical impact (CCI) device was used to establish the TBI model. Depression‐like behaviors in rats were assessed using the sucrose preference test (SPT), the tail suspension test (TST), and the forced swimming test (FST). The antioxidant system, lipid peroxidation, and ferroptosis levels were evaluated. The SLC7A11/GPX4 axis was analyzed using quantitative real‐time PCR (qRT‐PCR) and Western blot analysis.

**Results:**

Nicorandil administration significantly ameliorated depression‐like behaviors in rats with TBI. Nicorandil administration also effectively restored the antioxidant system, substantially reduced lipid peroxidation, and attenuated ferroptosis in the hippocampus of rats with TBI. Mechanistically, nicorandil administration promoted the SLC7A11/GPX4 axis in the hippocampus of rats with TBI. Crucially, knockdown of hippocampal SLC7A11 abrogated the protective effects of nicorandil on depression‐like behaviors, lipid peroxidation, and ferroptosis in the hippocampus of rats with TBI.

**Conclusion:**

These findings indicate that nicorandil ameliorates depression‐like behaviors following TBI by inhibiting hippocampal ferroptosis through the activation of the SLC7A11/GPX4 axis.

## Graphical abstract

Nicorandil administration significantly attenuates depression‐like behavior in rats with TBI. Mechanistically, nicorandil suppresses ferroptosis via promoting the SLC7A11/GPX4 axis in the hippocampus of rats with TBI, thus ameliorating depression‐like symptoms induced by TBI. Nicorandil: Nic.

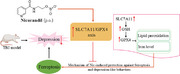

## 1 Introduction

Traumatic brain injury (TBI) is a complex neurological condition caused by an external mechanical force, potentially leading to a diminished or altered state of consciousness and profound psychological effects (Capizzi, Woo, and Verduzco‐Gutierrez 2020). Depression is one of the most frequent psychiatric outcomes following TBI, with approximately 30% of affected individuals developing post‐traumatic depression (Fakhoury et al. 2021). The pathogenesis of depression post‐TBI is multifaceted, involving inflammation, oxidative stress, mitochondrial dysfunction, blood–brain barrier disruption, and excitotoxicity (Boyko et al. 2023; Jahan and Tanev 2023). Despite ongoing research, the precise mechanisms underlying TBI‐related depression remain partially elucidated, and effective treatments to mitigate depression following TBI are not satisfactory.

Ferroptosis is a distinct form of programmed cell death characterized by the iron‐dependent accumulation of lipid peroxides to lethal levels (Jiang, Stockwell, and Conrad 2021). Recent research highlights the significant role of ferroptosis in the pathophysiology of TBI (Geng et al. 2021; Tang et al. 2020). Evidence demonstrates that ferroptosis is exacerbated in TBI, and its strategic inhibition can markedly attenuate hippocampal tissue damage, thereby improving long‐term outcomes after TBI, indicating ferroptosis as a contributing factor to the pathophysiology of TBI (Kenny et al. 2019; Rui et al. 2021; Xie et al. 2019). Furthermore, several studies also suggest a crucial role for ferroptosis in the development of depression (Dang et al. 2022; X. Wang, Li, et al. 2023). Pharmacological interventions targeting ferroptosis exhibit antidepressant effects (Jiao et al. 2021; E. Li et al. 2023; L. Wang, Xu, et al. 2023). Therefore, the increased susceptibility to ferroptosis in the brain may link directly to the onset of depression following TBI.

Nicorandil, a mitochondrial ATP‐sensitive potassium (mitoKATP) channel opener, has gained attention for its neuroprotective properties in various neurological diseases. It primarily functions by promoting antioxidative mechanisms, reducing inflammation, and enhancing mitochondrial function (Mustafa et al. 2024; Owjfard et al. 2024, 2021). In models of cerebral ischemia, nicorandil has been shown to improve neurological outcomes by activating KATP channels, which in turn limits oxidative damage and inflammatory response (Owjfard et al. 2024). Notably, our recent study reveals that nicorandil significantly attenuates the development of TBI (Tu et al. 2024), underscoring its potential as a therapeutic agent for TBI. In addition to oxidative stress and mitochondrial dysfunction, there is mounting evidence suggesting that ATP‐sensitive potassium (KATP) channels play a significant role in the pathophysiology of depression (Fan et al. 2016). Given this context, exploring the effects of nicorandil on depression following TBI and its underlying mechanisms may hold significant importance.

Numerous studies have established strong connections between oxidative stress, mitochondrial function, and ferroptosis (Yu et al. 2021). As nicorandil has well‐documented antioxidant property, we hypothesized that it may mitigate depression after TBI by attenuating hippocampal ferroptosis. A key mechanism in preventing ferroptosis involves the SLC7A11/glutathione peroxidase 4 (GPX4) (Chen, Li, et al. 2021), which enhances cystine uptake and promotes glutathione (GSH) synthesis, enabling GPX4 to inhibit lipid peroxidation and inhibit ferroptosis (Chen, Yu, et al. 2021). Given the significant role of ferroptosis in exacerbating post‐TBI complications, our study focuses on exploring whether nicorandil mitigates depression‐like behaviors in a rat model of TBI by activating the SLC7A11/GPX4 axis, thus reducing hippocampal ferroptosis.

## 2 Materials and Methods

### 2.1 Animals and Controlled Cortical Impact (CCI) Model

A total of 150 healthy adult male Sprague‐Dawley rats, weighing 280–320 g, aged 3–4 months, were acquired from the medical laboratory animal center of Nanchang University (Nanchang, China). The animals were housed under controlled environmental conditions, maintained on a 12‐h light–dark cycle with a temperature of 20°C–24°C and 40%–60% humidity levels. They had free access to water and food throughout the study period.

The TBI model was established using a CCI device (Hatteras Instruments Inc., Cary, NC, USA) as previously described (Ma et al. 2019). Briefly, rats were anesthetized with sodium pentobarbital (70 mg/kg) via intraperitoneal injection and positioned on a brain stereotaxis instrument. The surgical site on the head was disinfected with 70% ethanol, and a midline scalp incision was made. A craniotomy was performed using a bone drill to create a 5 mm diameter hole, positioned 3.5 lateral to the midline over the left hemisphere. Subsequently, a CCI device (PinPoint Model PCI3000 Precision Cortical Impactor, Hatteras Instruments, Cary, USA) was employed to induce a moderate TBI model, using general parameters of an impact depth of 1 mm, impact velocity of 3.0 m/s, and dwell time of 180 ms. Sham rats received a craniotomy except for CCI injury.

All experiments were conducted in accordance with the guide for the care and use of laboratory animals published by the US National Institutes of Health and were approved by the Ethics Committee of the First Affiliated Hospital of Zhengzhou University.

### 2.2 Experimental Design and Grouping

To investigate the protective effects of nicorandil on depression‐like behaviors following TBI and to elucidate the underlying mechanism, a total of 60 rats were randomly assigned to four groups (*n* = 15 per group): *Sham + Vehicle group*, rats underwent a single sham operation (craniotomy without CCI), and 1 h after surgery, they received daily oral administration of saline for 30 days; *TBI + Vehicle group*, rats underwent a single CCI procedure, and 1 h after surgery, they were orally administrated saline daily for 30 days; *TBI + Nicorandil group*, rats underwent a single CCI process, and 1 h after surgery, they were orally administrated nicorandil (7.5 mg/kg) daily for 30 days; and in *nicorandil group*, rats underwent a single sham operation, and 1 h after surgery, they were orally administrated with nicorandil (7.5 mg/kg) for 30 days.

To confirm the role of the SLC7A11/GPX4 axis in the protective effects of nicorandil against TBI, we used adeno‐associated viruses (AAV) carrying shRNA targeting SLC7A11 to selectively inhibit the SLC7A11/GPX4 axis in the hippocampus through stereotactic injection. A total of 90 rats were randomly assigned to six groups (*n* = 15 per group): Sham + Scramble shRNA group, TBI + Scramble shRNA group, TBI + Nicorandil + Scramble shRNA group, Sham + SLC7A11 shRNA group, TBI + SLC7A11 shRNA group, and TBI + Nicorandil + SLC7A11 shRNA group. A stereotaxic injection of either AAV‐Scramble shRNA or AAV‐SLC7A11 shRNA virus into the hippocampus was performed post‐CCI procedure.

On the day of the last administration of nicorandil, behavioral assays (open‐field test [OFT)] on day 30, sucrose preference test [SPT] on day 31, tail suspension test [TST] on day 34, and forced swimming test [FST] on day 35) were performed. After all behavior tests were conducted, rats were allowed to have rest for 1 day, and hippocampal tissue was collected to measure various experimental indicators.

### 2.3 Behavioral Tests

#### 2.3.1 SPT

The SPT is utilized to assess anhedonia, a core symptom of depression, by measuring the loss of interest in rewarding stimuli. During the adaptation phase, baseline sucrose preference was established by housing each rat individually in cages equipped with two bottles over a 12‐h period: one containing a 1% sucrose solution (weight/volume) and the other filled with an equivalent volume of pure water. Subsequently, rats (*n* = 15 per group) underwent a 12‐h period of food and water deprivation to ensure a strong motivational state for the test phase. Following deprivation, each rat was given continuous access to both the sucrose solution and water for an additional 12 h. To control for side preference, the positions of the bottles were switched at the 6‐h mark. The sucrose preference index was calculated as follows: Sucrose Preference Index (%) = (g)/[sucrose intake (g) + water intake (g)] × 100%.

#### 2.3.2 TST

The TST is a widely used behavioral assay to evaluate depression‐like behaviors in rodents. In this test, each rat was gently restrained while a nonstick adhesive tape was placed one‐third of the way from the tip of the tail. The rat was then suspended by this segment of the tail using a clip attached to a stand, ensuring the rat's head was maintained approximately 10 cm above the floor. The entire progression from active struggling to cessation of movement and finally to immobility was documented by the camera, all occurring within a span of 6 min. The duration of immobility is quantified and used as a primary measure of the severity of depression‐like symptoms.

#### 2.3.3 OFT

The OFT is a commonly employed behavioral assay in animal studies, designed to assess exploratory behavior, general activity levels, and anxiety‐like behaviors in rodents. Briefly, rats were placed at the center of an open‐box device (dimensions: 80 × 80 × 40 cm^3^), which is divided into 16 equal squares. Each rat was allowed to explore the arena freely for 5 min. During this time, the activity metrics, including the total distance moved and the time spent in the central area, were tracked using an automated system to assess levels of exploration and anxiety. After each session, the bottom of the apparatus was sanitized with a low‐concentration ethanol solution to eliminate olfactory cues and ensure standard conditions for each test.

#### 2.3.4 FST

The FST is a well‐established method for evaluating depressive‐like behaviors in rodents. During the test, each rat was placed in a cylindrical container (50 cm in height and 20 cm in diameter) filled with water (up to 30 cm high) at a specific temperature approximately 25°C. The test typically lasts for 6 min, with the first 2 min considered as an acclimatization period. Immobility time, defined as the period during which the animal remains floating with minimal movements to keep its head above water, was recorded during the last 4 min.

### 2.4 Assessment of Lipid Peroxidation Levels

The malondialdehyde (MDA) level, superoxide dismutase (SOD) activity, and GSH content in the hippocampus tissues were, respectively, determined using an MDA assay kit (Beyotime Institute of Biotechnology, Nantong, China), SOD assay kit (Jiancheng Bioengineering Institute, Nanjing, China), and GSH assay kit (Abcam, Cambridge, UK) following the standard protocol.

### 2.5 Iron Content Assay

The intracellular iron concentration in the supernatants from lysed hippocampal tissues was determined using an iron assay kit (Jiancheng Bioengineering Institute, Nanjing, China) according to the standard manufacturer's procedures. After behavioral testing, hippocampus tissues were collected and homogenized in RIPA lysis buffer containing phenylmethylsulfonyl fluoride (PMSF; 10 µM) using a mechanical homogenizer. After centrifugation at 12,000 *g* for 15 min at 4°C, the supernatants were carefully collected. The protein concentrations were quantified using the bicinchoninic acid (BCA) assay (Beyotime Institute of Biotechnology, Nantong, China). The protein extracts were stored at −80°C until analysis. To measure iron concentration, the supernatant samples and standards were added to the appropriate wells of a microplate. Then, 5 µL of iron‐reducing agent was added and incubated for 30 min at room temperature in darkness. The absorbance at 593 nm was evaluated using a microplate reader (Synergy HT, BioTek, VT, USA).

### 2.6 Quantitative Real‐Time Polymerase Chain Reaction (qRT‐PCR) Analysis

Total RNA was extracted from the hippocampal tissue using Trizol Reagent (Sigma, USA). Approximately 1 µg of RNA was then reverse transcribed into cDNA using a Prime‐Script RT reagent kit (Servicebio, Wuhan, Hubei, China). The qRT‐PCR was performed using a SYBR Green Master Mix (TaKaRa, Japan) on an ABI PRISM 7500 system (Applied BioSystems, Waltham, MA, USA). The mRNA expression levels were normalized to a housekeeping gene (β‐actin) using the 2^−ΔΔCt^ method. The sequences of the primers were listed as follows:

IL‐1β Forward: 5′‐ ATCTCACAGCATCTCGACAAG‐3′,
Reverse 5′‐CACACTAGCAGGTCGTCATCC‐3′;
IL‐6 Forward: 5′‐ AGGAGTGGCTAAGGACCAAGACC‐3′,
Reverse 5′‐ TGCCGAGTAGACCTCATAGTGACC‐3′;
TNF‐α Forward: 5′‐ GCATGATCCGAGATGTGGAACTGG‐3′,
Reverse 5′‐ CGCCACGAGCAGGAATGAGAAG‐3′;
GPX4 Forward: 5′‐ CGATACGCTGAGTGTGGTTT‐3′,
Reverse 5′‐ CGGCGAACTCTTTGATCTCTT‐3′;
SLC7A11 Forward: 5′‐ GGTTGCCCTTTCCCTCTATTC‐3′,
Reverse 5′‐CCTGGGTTTCTTGTCCCATATAA‐3′;
FTH1 Forward: 5′‐ TACCTGAATGAGCAGGTGAAAG‐3′,
Reverse 5′‐GATATTCCGCCAAGCCAGAT‐3′;
PTGS2 Forward: 5′‐CTTCGGGAGCACAACAGAGT‐3′;
Reverse 5′‐TTCAGAGGCAATGCGGTTCT‐3′;
β‐actin Forward: 5′‐CACGATGGAGGGGCCGGACTCATC‐3′,
Reverse 5′‐ TAAAGACCTCTATGCCAACACAGT‐3′.

### 2.7 Western Blot Analysis

Total protein was extracted from the hippocampal tissue using RIPA lysis buffer (Beyotime, Shanghai, China) containing protease and phosphatase inhibitors (Beyotime) on ice for 30 min. Protein concentration was determined by a BCA protein assay kit (Thermo Scientific). Equal amounts of proteins were separated on a 10% SDS‐PAGE gel and then transferred to polyvinylidene difluoride (PVDF) membranes. The membranes were then blocked in Tris‐buffered saline containing Tween‐20 (TBST) with 5% nonfat milk for 1 h at room temperature and incubated with primary antibodies at 4°C with shaking overnight. The primary antibodies used were as follows: SLC7A11 (1:1000, Cell Signaling Technology, Danvers, MA, USA), GPX4 (1:1000, Cell Signaling Technology), and GAPDH (1:4000, Cell Signaling Technology). After washing three times with TBST, the membranes were further incubated with peroxidase‐labeled secondary antibodies (1:5000, Santa Cruz Biotechnology, Dallas, TX, USA) at room temperature for 1 h. The immunoblotting bands were developed using enhanced chemiluminescence (ECL) solution (Genesee Scientific, San Diego, CA, USA). The gray values of the protein bands were quantified with ImageJ software (NIH, Bethesda, MD, USA).

### 2.8 Statistical Analysis

All data were analyzed using SPSS (version 26.0; IBM, Armonk, NY) and are represented as the mean ± standard deviation (SD). Group differences were conducted using one‐way ANOVA, followed by Tukey's post hoc multiple comparisons. *p* < 0.05 was considered statistically significant.

## 3 Results

### 3.1 Nicorandil Improves Depression‐Like Behaviors in Rats With TBI

**FIGURE 1:**
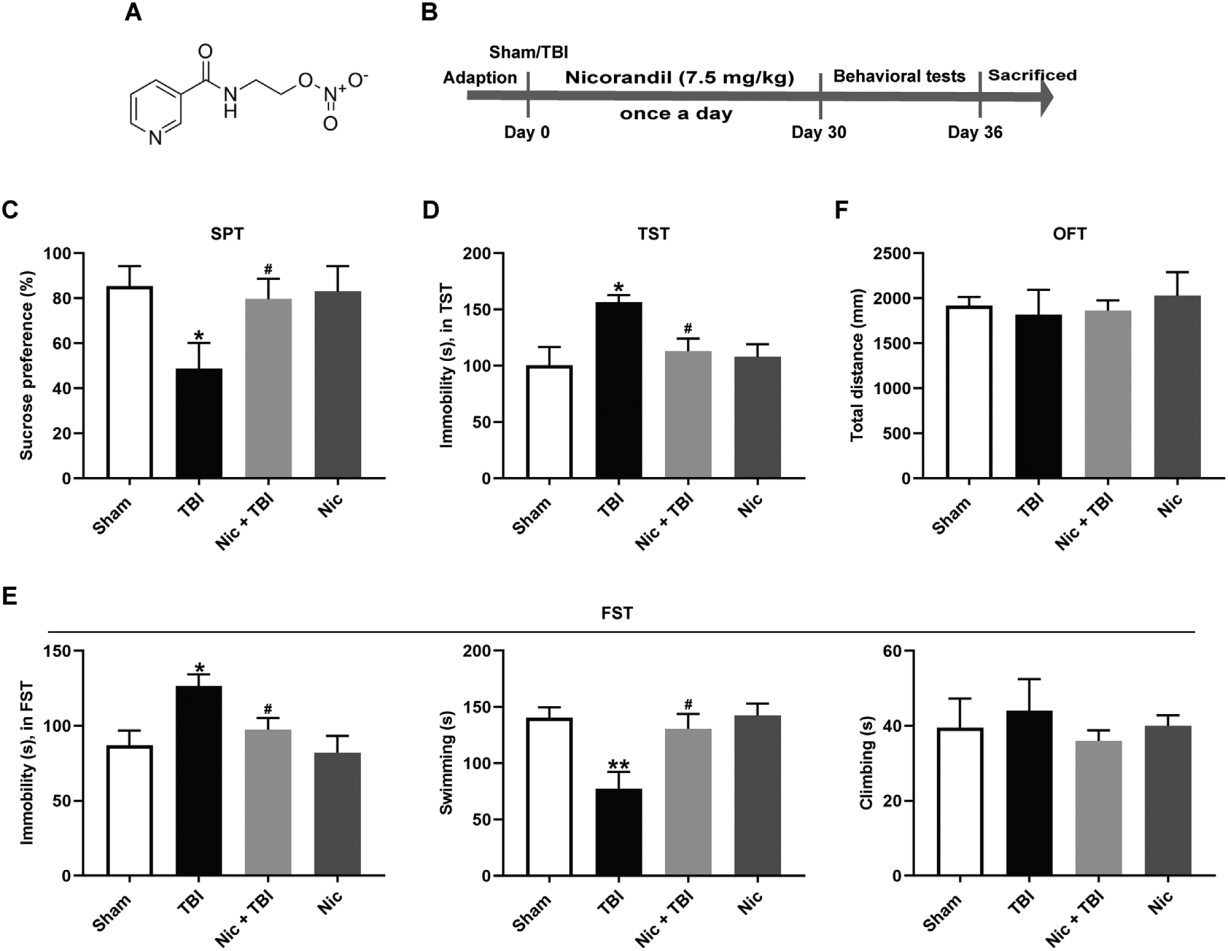
Effects of nicorandil on depression‐like behaviors in rats with TBI. (A) The chemical structure of nicorandil. (B) Schematic timeline of the experimental procedures. (C) Results from the sucrose preference test (SPT) illustrate a significant reduction in sucrose preference in TBI rats compared to controls; nicorandil treatment significantly elevated sucrose preference in rats with TBI. (D) Tail suspension test (TST) results show increased immobility in rats with TBI, which was reduced by nicorandil administration. (E) Open‐field test (OFT) outcomes show no significant differences in distance traveled or time spent in the center across all groups. (F) Forced swimming test (FST) results indicate increased immobility and decreased swimming in rats with TBI, which were ameliorated by nicorandil administration. The data are expressed as means ± SEM (*n* = 8–10). ^*^ *p* < 0.05, ^**^ *p* < 0.01 vs. the scramble group; ^#^ *p* < 0.05 vs. the TBI group.

To assess the effects of nicorandil on neurological deficits following TBI, we conducted several behavioral tests, including the SPT, the TST, and the FST (Figure 1A,B). As shown in Figure 1C, rats subjected to TBI exhibited a significant reduction in sucrose preference compared to the scramble group (*p* < 0.05). However, nicorandil administration significantly increased sucrose preference in rats with TBI (*p* < 0.05). In addition, rats with TBI exhibited substantial increased immobility time during the TST (Figure 1D, *p* < 0.05), suggesting depressive‐like states. In contrast, nicorandil treatment significantly reduced the immobility in rats with TBI (Figure 1D, *p* < 0.05). To rule out the influence of the locomotion and exploratory behavior, an OFT was carried out. The results showed no differences in the distance traveled and time in the center among all groups (Figure 1E, *p* > 0.05). Moreover, in the FST, rats with TBI showed increased immobility (Figure 1F, *p* < 0.05) and decreased swimming time (Figure 1F, *p* < 0.01), both of which were mitigated by nicorandil treatment (Figure 1F, *p* < 0.05). There were no differences in climbing behavior among the four groups (Figure 1F, *p* > 0.05). Collectively, these results indicate that nicorandil effectively ameliorates depressive‐like behaviors in rats following TBI.

### 3.2 Nicorandil Inhibits Ferroptosis in the Hippocampus of Rats With TBI

**FIGURE 2:**
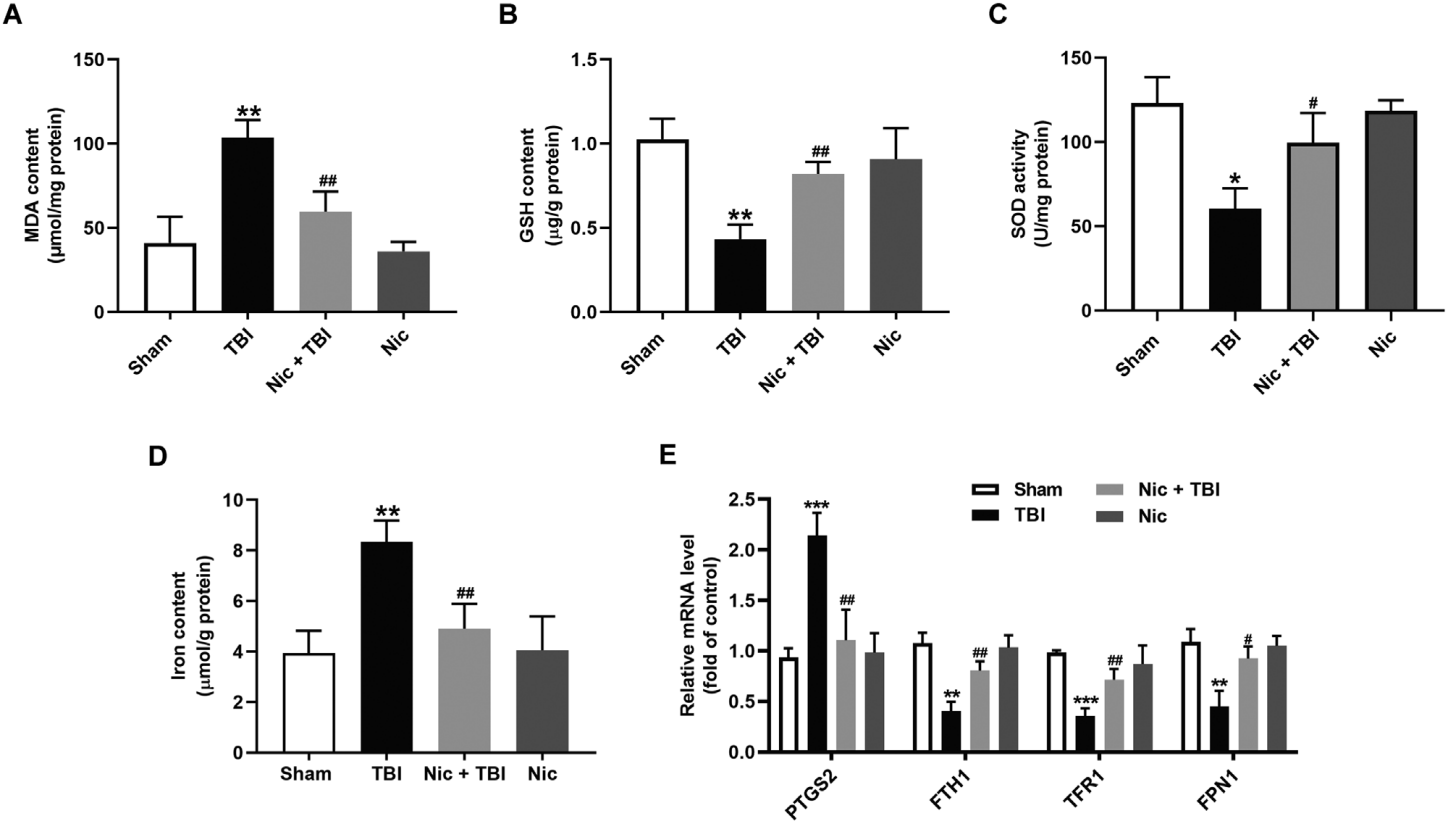
Effects of nicorandil on ferroptosis in the hippocampus of rats with TBI. (A) Malondialdehyde (MDA) level in the hippocampus, showing a significant increase in rats with TBI compared to the scramble group, and a significant reduction following nicorandil administration. (B) Glutathione (GSH) content in the hippocampus, showing a significant decrease in rats with TBI that was restored by nicorandil treatment. (C) Superoxide dismutase (SOD) activity in the hippocampus, with trends similar to GSH content. (D) Iron level in the hippocampus, showing an increase in rats with TBI and a deduction in nicorandil administration. (E) The mRNA levels of PTGS2, FTH1, TFR1, and FPN1 in the hippocampus, showing significant alterations in TBI rats, all of which were reversed by nicorandil administration. Data are presented as mean ± SEM (*n* = 8–10). ^*^ *p* < 0.05, ^**^ *p* < 0.01, ^***^ *p* < 0.001 vs. the scramble group; ^#^ *p* < 0.05, ^##^ *p* < 0.01 vs. the TBI group.

Ferroptosis, characterized by lipid peroxidation, is a crucial pathological process in the pathophysiology of TBI (Gao et al. 2023; Xie et al. 2019). To explore whether the antidepressive effects of nicorandil following TBI are associated with its regulation of ferroptosis, we determined the effects of nicorandil on MDA, a key biomarker of lipid peroxidation, in the hippocampus. Our findings demonstrated that the level of MDA was significantly elevated in the hippocampus of rats with TBI (Figure 2A, *p* < 0.01), while nicorandil administration significantly reduced the level of MDA in the hippocampus of rats with TBI (Figure 2A, *p* < 0.01). Additionally, we assessed the effects of nicorandil on antioxidant enzymes, including GSH and SOD. Both the content of GSH (Figure 2B, *p* < 0.01) and the activity of SOD (Figure 2C, *p* < 0.05) were notably suppressed in the hippocampus of rats with TBI, and these reductions were blocked by nicorandil administration. Furthermore, we assessed iron accumulation, another hallmark of ferroptosis. The level of iron was significantly increased in the hippocampus of rats with TBI (Figure 2D, *p* < 0.01), while nicorandil administration remarkably reduced the level of iron in the hippocampus of rats with TBI (Figure 2E, *p* < 0.01). qRT‐PCR analysis revealed that the mRNA level of prostaglandin‐endoperoxide synthase 2 (PTGS2, *p* < 0.001), responsible for iron storage, was increased in the hippocampus of rats with TBI. Conversely, the mRNA levels of ferritin heavy chain 1 (FTH1, *p* < 0.01), transferrin receptor 1 (TFR1, *p* < 0.001), and ferroportin 1 (FPN1, *p* < 0.01), all involved in iron import, were decreased in the hippocampus of rats with TBI (Figure 2F). However, these effects were all blocked by nicorandil administration. Taken together, these results suggest that nicorandil alleviates ferroptosis in the hippocampus, which may contribute to its antidepressive effects in TBI.

### 3.3 Nicorandil Promotes the SLC7A11/GPX4 Axis in the Hippocampus of Rats With TBI

**FIGURE 3:**
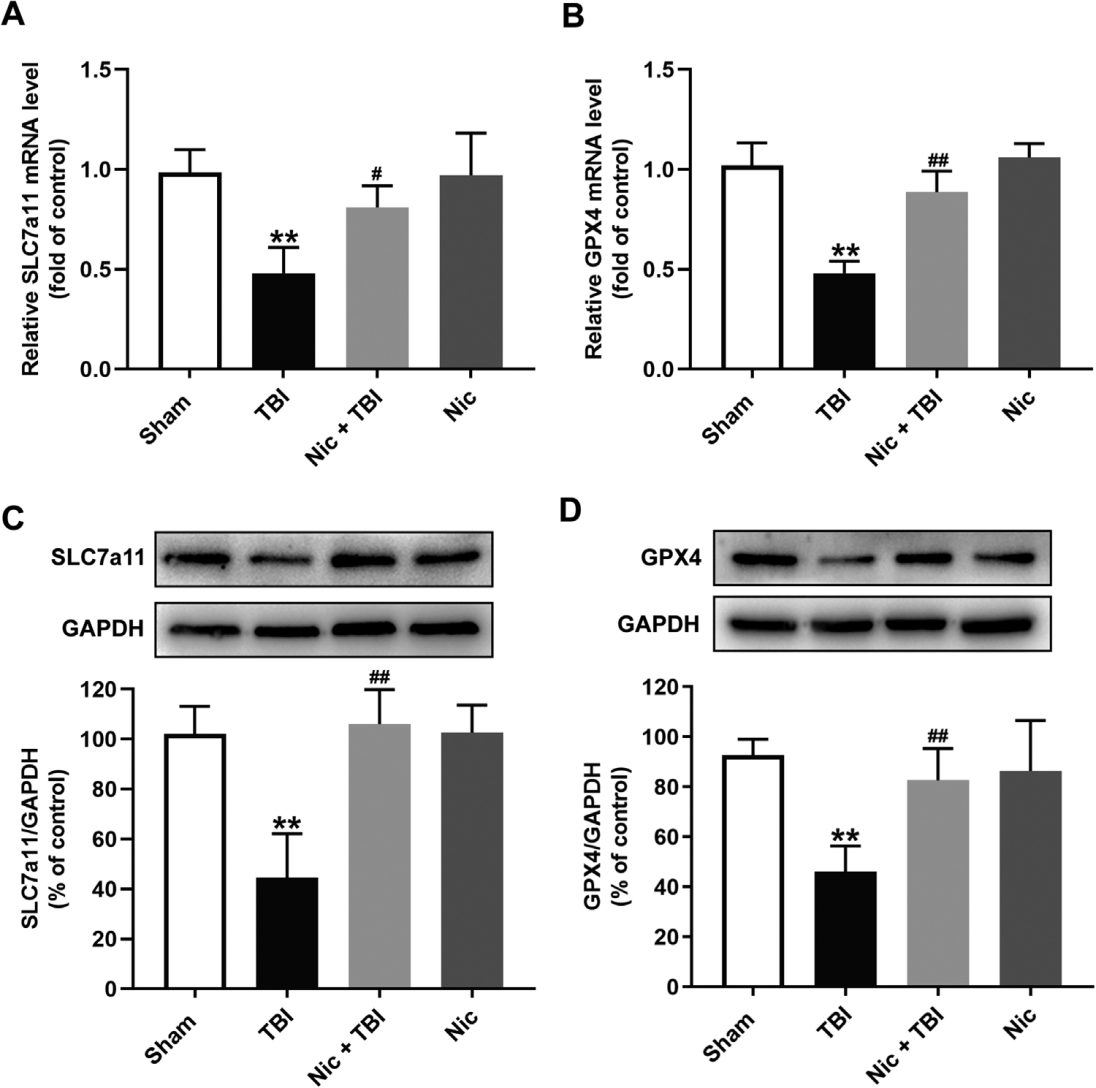
Effects of nicorandil on the SLC7A11/GPX4 axis in the hippocampus of rats with TBI. (A) QRT‐PCR analysis of SLC7A11 mRNA level and (B) Western blot analysis of SLC7A11 protein expression in the hippocampus, showing significant reductions in in the hippocampus of rats with TBI compared to the control group, with restoration following nicorandil administration. (C) QRT‐PCR analysis of GPX4 mRNA level and (D) Western blot analysis of GPX4 protein expression in the hippocampus, demonstrating similar trends as SCL7A11 levels. Data are expressed as means ± SEM (*n* = 4–6). ^**^ *p* < 0.01 vs. the scramble group; *^#^p* < 0.05, ^##^ *p* < 0.01 vs. the TBI group.

To elucidate the mechanisms underlying the antidepressive effects of nicorandil in TBI, we analyzed the impact of nicorandil on the SLC7A11/GPX4 axis, utilizing both qRT‐PCR and Western blot analysis. We observed significantly lower mRNA and protein levels of SLC7A11 (Figure 3A,B, *p* < 0.01) and GPX4 (Figure 3C,D, *p* < 0.01) in the hippocampus of rats with TBI. Nicorandil treatment effectively restored the mRNA and protein levels of SLC7A11 (Figure 3A,B, *p* < 0.01) and GPX4 (Figure 3C,D, *p* < 0.01) in the hippocampus of rats with TBI. These results suggest that nicorandil may exert its protective effects against TBI‐induced depression by activating the SLC7A11/GPX4 axis in the hippocampus.

### 3.4 Hippocampal SLC7A11 Knockdown Attenuates the Antidepressant Effect of Nicorandil Following TBI

**FIGURE 4:**
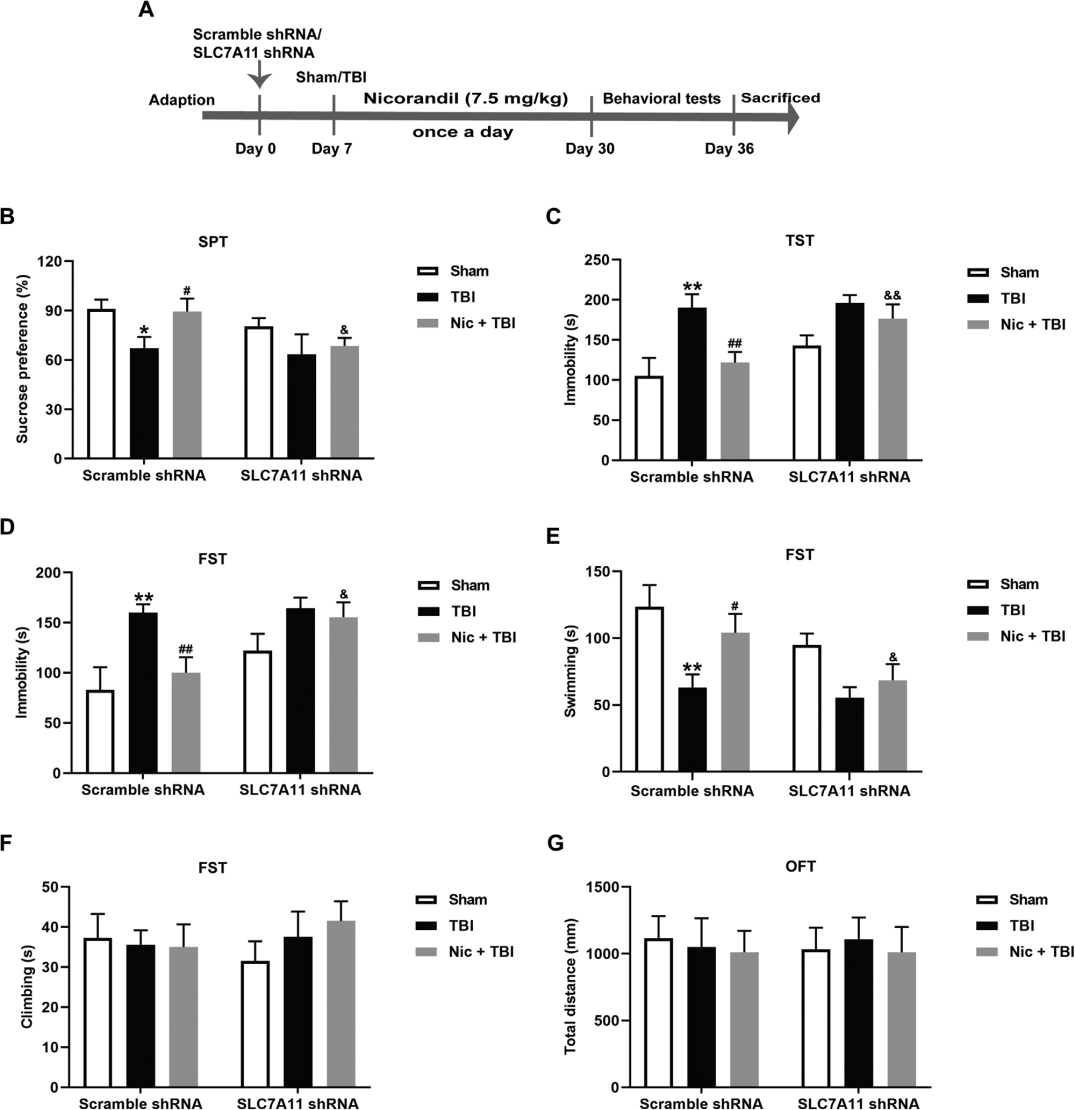
Effects of hippocampal SLC7A11 knockdown on the protective effects of nicorandil on the depression‐like behaviors in rats with TBI. (A) Schematic timeline of the experimental procedures. (B) QRT‐PCR analysis show a significant reduction in SLC7A11 and GPX4 mRNA levels in the hippocampus of rats treated with nicorandil post‐TBI after hippocampal SLC7A11 shRNA injection. (C) SPT results indicate a decreased sucrose preference in rats with hippocampal SLC7A11 knockdown compared to the nicorandil treatment following TBI. (D) TST results show an increased immobility in rats with hippocampal SLC7A11 knockdown. (E–G) FST results show increased immobility, decreased swimming, and no changed climbing in rats with hippocampal SLC7A11 knockdown. (G) OFT results demonstrate no significant differences in distance traveled or time spent in the center among all groups. The data are expressed as means ± SEM (*n* = 8–10). ^*^ *p* < 0.05, ^**^ *p* < 0.01 vs. the scramble group; ^#^ *p* < 0.05, ^##^ *p* < 0.01 vs. the TBI group; ^&^ *p* < 0.05, ^&&^ *p* < 0.01 vs. the nicorandil + TBI group.

To further validate the role of the SLC7A11/GPX4 axis in the antidepressant efficacy of nicorandil following TBI, rats were injected with SLC7A11 shRNA to suppress the SLC7A11/GPX4 axis, and subsequent depression‐like behaviors were detected (Figure 4A). As expected, SLC7A11 shRNA injection significantly reduced the mRNA levels of SLC7A11 and GPX4 in the hippocampus of rats treated with nicorandil following TBI (Figure 4B, *p* < 0.01). Knockdown of hippocampal SLC7A11 significantly decreased sucrose preference compared to that in rats treated with nicorandil following TBI (Figure 4C, *p* < 0.05). In addition, knockdown of hippocampal SLC7A11 remarkably attenuated nicorandil administration‐induced decrease in the immobility of rats with TBI in the TST (Figure 4D, *p* < 0.01). Moreover, knockdown of hippocampal SLC7A11 also reduced the nicorandil administration‐induced the decrease in the immobility (Figure 4E, *p* < 0.05) and an increase in swimming (Figure 4F, *p* < 0.05) in rats with TBI. The climbing among groups had no changes (Figure 4G, *p* > 0.05). OFT results showed that there were no differences in the distance traveled and time in the center among all groups (Figure 4H, *p* > 0.05). These results indicate that inhibition of the hippocampal SLC7A11/GPX4 axis blocks the protective effect of nicorandil on depression following TBI.

### 3.5 Hippocampal SLC7A11 Knockdown Blocks the Inhibition of Nicorandil on Ferroptosis in the Hippocampus of Rats With TBI

**FIGURE 5:**
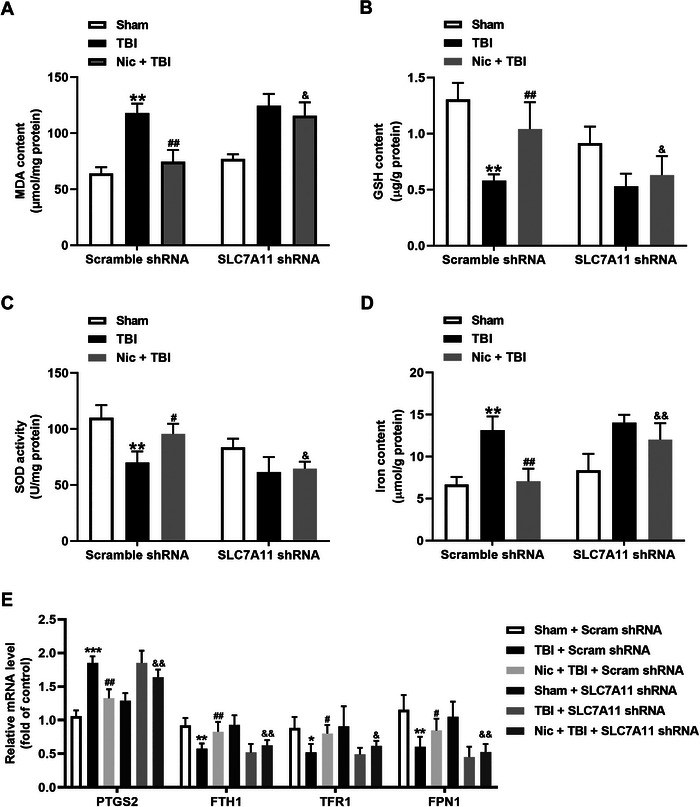
Effects of hippocampal SLC7A11 knockdown on the protective effects of nicorandil on ferroptosis in the hippocampus of rats with TBI. (A) Level of MDA in the hippocampus, showing a significant increase following SLC7A11 knockdown in nicorandil and TBI co‐treated rats. (B) Content of GSH in the hippocampus, showing significant reduction following SLC7A11 knockdown in nicorandil and TBI co‐treated rats. (C) Superoxide dismutase (SOD) activity in the hippocampus, with similar trends as GSH content. (D) Level of iron in the hippocampus, showing significant increase following SLC7A11 knockdown in nicorandil and TBI co‐treated rats. (E) The mRNA levels of PTGS2, FTH1, TFR1, and FPN1 in the hippocampus. Data are expressed as means ± SEM (*n* = 8–10). ^*^ *p* < 0.05, ^**^ *p* < 0.01, ^***^ *p* < 0.001, vs. the scramble group; ^#^ *p* < 0.05, ^##^ *p* < 0.01 vs. the TBI group; ^&^ *p* < 0.05, ^&&^ *p* < 0.01 vs. the nicorandil + TBI group.

Finally, we explored the role of the SLC7A11/GPX4 axis in nicorandil‐reduced ferroptosis following TBI. As shown in Figure 5, knockdown of hippocampal SLC7A11 obviously increased the levels of MDA in the hippocampus of rats treated with nicorandil following TBI (Figure 5A, *p* < 0.05). In addition, knockdown of hippocampal SLC7A11 mitigated nicorandil‐promoted antioxidative defense in the hippocampus of rats treated with TBI, as evidenced by the decreases in the content of GSH (Figure 5B, *p* < 0.05) and the activity of SOD (Figure 5C, *p* < 0.05). Furthermore, knockdown of hippocampal SLC7A11 increased iron levels in the hippocampus of rats treated with nicorandil following TBI (Figure 5D, *p* < 0.01). qRT‐PCR analysis results revealed that knockdown of hippocampal SLC7A11 led to an increased level of PTGS2 (*p* < 0.01) and the decreased levels of FTH1 (*p* < 0.01), TFR1 (*p* < 0.05), and FPN1 (*p* < 0.01) in the hippocampus of rats treated with nicorandil following TBI (Figure 5E). These results indicated that inhibition of the hippocampal SLC7A11/GPX4 axis alleviates the protection of nicorandil against ferroptosis following TBI.

## 4 Discussion

Our previous study has established the efficacy of nicorandil in attenuating the neurological deficits associated with TBI (Tu et al. 2024); however, the effects of nicorandil on depression following TBI have not been explored. In this study, we identified that nicorandil administration significantly attenuates depression‐like behavior in rats with TBI. Mechanistically, our results suggest that nicorandil exerts its antidepressant effects during TBI by suppressing ferroptosis through promoting the SLC7A11/GPX4 axis in the hippocampus. These findings highlight nicorandil as a promising therapeutic candidate for addressing the common and debilitating effects of depression associated with TBI and open new avenues for the treatment of depression in TBI patients.

Depression is a common sequela following TBI, affecting approximately 30%–50% of TBI patients within the first year postinjury. Managing post‐TBI depression presents a significant clinical challenge (Howlett, Nelson, and Stein 2022; Maas et al. 2022). Nicorandil, a well‐known vasodilatory drug primarily used to treat angina, has emerged as a potential neuroprotective agent. Its ability to activate mitochondrial K_ATP channels and reduce oxidative stress has been shown to play a critical role in mitigating various forms of neuronal damage (Ahmed and El‐Maraghy 2013; Ravindran et al. 2017). Both mitochondrial dysfunction and oxidative stress often contribute to neuropsychiatric disorders, including TBI and such as depression (Bansal and Kuhad 2016; Bhatt, Nagappa, and Patil 2020; J. Li et al. 2022). Our previous study reveals that nicorandil attenuates cognitive impairment after TBI through suppressing oxidative stress and inflammation (Tu et al. 2024). Hence, we have reason to believe that nicorandil may improve depression following TBI. Indeed, our current study is the first to demonstrate that nicorandil administration significantly attenuates depression‐like behavior in rats with TBI, suggesting its potential utility in treating depression among TBI patients.

Ferroptosis is a form of programmed cell death characterized by the accumulation of iron‐dependent lipid peroxides (Jiang, Stockwell, and Conrad 2021). It is increasingly recognized for its role in various neuropathological conditions, including TBI (Gao et al. 2023; Geng et al. 2021). Studies have shown that ferroptosis is significantly upregulated during TBI, as evidenced by an increase in MDA level and the decreases in the SOD and GSH‐PX activities (Gao et al. 2023). The hippocampus, a region critical for emotional regulation and cognitive functions, is particularly vulnerable to oxidative stress and subsequent ferroptotic cell death in the context of TBI (Fang et al. 2023; Kenny et al. 2019). Consistent with these studies, our findings reveal that TBI leads to lipid peroxidation, reduces antioxidant defense, and increases ferroptosis in the hippocampus of rats. Emerging research indicates a significant link between ferroptosis and the pathophysiology of depression (X. Wang, Li et al. 2023; Z. Wang et al. 2022). Inhibiting ferroptosis has been shown to attenuate depression (Dang et al. 2022; E. Li et al. 2023). Several pharmacological agents and approaches targeting ferroptosis pathways have been explored for the potential treatment of depression (Dang et al. 2022; Jiao et al. 2021; R. Yang et al. 2023). Interestingly, our findings showed that nicorandil administration attenuates lipid peroxidation and ferroptosis in the hippocampus of rats with TBI. Hence, the above findings indicate that nicorandil attenuates ferroptosis, thereby potentially suppressing depression after following TBI.

SLC7A11, a component of the cystine/glutamate antiporter system x_c^‐, regulates cystine uptake and glutamate release (Dahlmanns et al. 2023). Its activation leads to increased intracellular cystine, which is subsequently converted to GSH, a major antioxidant. GPX4 utilizes GSH to reduce lipid hydroperoxides, thereby preventing ferroptotic cell death (Imai et al. 2017). In the context of depression, activation of the SLC7A11/GPX4 axis might mitigate oxidative stress and lipid peroxidation, reducing ferroptosis and alleviating depressive symptoms (Z. Yang et al. 2024; Zhou et al. 2024). This suggests that targeting the SLC7A11/GPX4 axis to regulate ferroptosis may offer new therapeutic strategies for TBI‐related depression. Interestingly, our study found that nicorandil administration promotes the SLC7A11/GPX4 axis in the hippocampus of rats with TBI. Knockdown of hippocampal SLC7A11 attenuates the antidepressant effects of nicorandil following TBI. This finding indicates that the SLC7A11/GPX4 axis is not only crucial for controlling ferroptosis but also integral to the antidepressant efficacy of nicorandil during TBI. Moreover, SLC7A11 knockdown blocks the nicorandil‐induced reduction in ferroptosis, further supporting the hypothesis that the therapeutic benefits of nicorandil in TBI‐related depression are mediated through its anti‐ferroptotic actions. Further research should aim to delineate the detailed mechanisms by which nicorandil modulates the SLC7A11/GPX4 axis and to explore the potential clinical applications of this pathway in human subjects with TBI.

Several limitations warrant consideration. First, nicorandil is a potassium channel agent that can promote nitric oxide (NO) production (Ahmed 2019). Future research is necessary to determine whether potassium channels and the NO pathway contribute to the protection of nicorandil against depression following TBI. Second, various forms of cell death—such as apoptosis, ferroptosis, autophagy, and necroptosis—are implicated in depression related to TBI (Maas et al. 2022); however, we did not discuss how much level of ferroptosis contributes to this condition. SLC7A11 functions as a key component of the cystine/glutamate antiporter system and confers specificity for cystine uptake (Koppula et al. 2018). Third, a more thorough evaluation, including transport assays or the use of SLC7A11 inhibitors (e.g., sulfasalazine), is necessary to confirm whether nicorandil influences the transport activity of SLC7A11 in future research. Finally, the focus of this study is limited to the SLC7A11/GPX4 axis, which is just one of many signaling pathways involved in ferroptosis. A comprehensive exploration of additional pathways may provide a more nuanced understanding of the mechanisms underlying the therapeutic effects of nicorandil in TBI‐related depression.

Taken together, our results reveal that nicorandil attenuates depression following TBI via facilitating the SLC7A11/GPX4 axis to suppress hippocampal ferroptosis. These findings suggest that nicorandil could be a promising therapeutic strategy for the development of treatments targeting depression associated with TBI.

## Author Contributions

**Yao‐Ran Tu**: methodology, investigation, validation, writing–original draft. **Ming Tan**: methodology, data curation. **Yao Li**: methodology, data curation. **De‐Quan Hong**: methodology, investigation. **Fan Niu**: formal analysis, writing–original draft, writing–review and editing, methodology.

## Conflicts of Interest

The authors declare no conflicts of interest.

### Peer Review

The peer review history for this article is available at https://publons.com/publon/10.1002/brb3.70199

## Data Availability

All data generated or analyzed during this study are included in this published article.
